# From ionic to cellular variability in human atrial myocytes: an integrative computational and experimental study

**DOI:** 10.1152/ajpheart.00477.2017

**Published:** 2017-12-22

**Authors:** Anna Muszkiewicz, Xing Liu, Alfonso Bueno-Orovio, Brodie A. J. Lawson, Kevin Burrage, Barbara Casadei, Blanca Rodriguez

**Affiliations:** ^1^Department of Computer Science, University of Oxford, Oxford, United Kingdom; ^2^Division of Cardiovascular Medicine, Radcliffe Department of Medicine, University of Oxford, John Radcliffe Hospital, Oxford, United Kingdom; ^3^ARC Centre of Excellence for Mathematical and Statistical Frontiers, School of Mathematical Sciences, Queensland University of Technology, Brisbane, Queensland, Australia; ^4^School of Mathematics, Queensland University of Technology, Brisbane, Queensland, Australia

**Keywords:** action potential, population of models

## Abstract

Variability refers to differences in physiological function between individuals, which may translate into different disease susceptibility and treatment efficacy. Experiments in human cardiomyocytes face wide variability and restricted tissue access; under these conditions, computational models are a useful complementary tool. We conducted a computational and experimental investigation in cardiomyocytes isolated from samples of the right atrial appendage of patients undergoing cardiac surgery to evaluate the impact of variability in action potentials (APs) and subcellular ionic densities on Ca^2+^ transient dynamics. Results showed that *1*) variability in APs and ionic densities is large, even within an apparently homogenous patient cohort, and translates into ±100% variation in ionic conductances; *2*) experimentally calibrated populations of models with wide variations in ionic densities yield APs overlapping with those obtained experimentally, even if AP characteristics of the original generic model differed significantly from experimental APs; *3*) model calibration with AP recordings restricts the variability in ionic densities affecting upstroke and resting potential, but redundancy in repolarization currents admits substantial variability in ionic densities; and *4*) model populations constrained with experimental APs and ionic densities exhibit three Ca^2+^ transient phenotypes, differing in intracellular Ca^2+^ handling and Na^+^/Ca^2+^ membrane extrusion. These findings advance our understanding of the impact of variability in human atrial electrophysiology.

**NEW & NOTEWORTHY** Variability in human atrial electrophysiology is investigated by integrating for the first time cellular-level and ion channel recordings in computational electrophysiological models. Ion channel calibration restricts current densities but not cellular phenotypic variability. Reduced Na^+^/Ca^2+^ exchanger is identified as a primary mechanism underlying diastolic Ca^2+^ fluctuations in human atrial myocytes.

## INTRODUCTION

Investigations of cardiomyocyte electrical properties in animal models can be controlled to limit variability; in contrast, heterogeneity in the human population and representativeness of the myocytes obtained from cardiac biopsies are key challenges in our understanding of human cardiac physiology. Ion current density can also vary in response to intracellular and external stimuli. In particular, ionic current properties are modulated by signaling molecules such as nitric oxide (13, 14, 36), hormones (1, 2, 47), nutrients (57), the circadian rhythm (19), and temperature (10, 11). Therefore, when investigating cardiac physiology, we are facing a moving target that is modulated by a host of internal and external factors.

A further constraint in the characterization of human physiology is limited access to human tissue. For this reason, data sets in cardiac electrophysiology often consist of standard action potential (AP) measurements at a single frequency; sometimes only biomarker values, such as the AP duration (APD), are available (18, 20, 35, 39). Similarly, ionic currents are frequently characterized using voltage-clamp recordings in cells that are different from those used for AP measurements, reflecting poor survival of cells subjected to different experimental configurations, solutions, and pharmacological action. Experimental electrophysiological recordings therefore offer a valuable but restricted snapshot of specific properties in isolated tissue/cells. Importantly, integration of these resources remains an open challenge (38).

Construction of biophysically detailed models and simulations can help integrating experimental data and improving knowledge of human electrophysiology (5, 27). Recently, consideration of biological variability has triggered further investigations into its underlying ionic basis and implications for disease and treatment (25, 29, 40, 54). However, previous studies of variability in human atrial electrophysiology only had access to AP biomarkers, measured in heterogeneous patient cohorts (20, 39).

Here, we investigated how variability in specific ionic currents impacts the phenotypic variability observed in human atrial APs and Ca^2+^ transients using three different and well-established models of human atrial electrophysiology. To do so, we used a rich ex vivo data set consisting of APs recorded at five pacing frequencies in human right atrial myocytes in addition to measurements of four key currents underlying atrial plateau and repolarization phases [namely, the transient outward K^+^ current (*I*_to_), inward rectifier K^+^ current (*I*_K1_), L-type Ca^2+^ current (*I*_CaL_), and atrial-specific ultrarapid K^+^ current (*I*_Kur_)] in a controlled cohort of patients under sinus rhythm.

Our results support a wide variability in ionic current densities for both K^+^ and Ca^2+^ currents in human atrial cardiomyocytes. In addition to the role of *I*_CaL_ in modulating total intracellular Ca^2+^, our findings suggest that differences in human atrial Ca^2+^ transients are primarily driven by intracellular Ca^2+^-handling and Ca^2+^ membrane extrusion. By exploiting the constructed populations of human atrial models, we demonstrate that diastolic Ca^2+^ fluctuations are determined not only by ryanodine receptor (RyR) or sarco(endo)plasmic reticulum Ca^2+^-ATPase (SERCA), as previously reported (33), but importantly also by the Na^+^/Ca^2+^ exchanger (NCX). By integrating experiments and simulations, we extend the existing knowledge on ionic and subcellular mechanisms underpinning variability in human atrial electrophysiology, with important implications for further understanding the mechanisms of intracellular atrial Ca^2+^ dysregulation.

## METHODS

### 

#### Experimental data set.

**Fig. 1.**
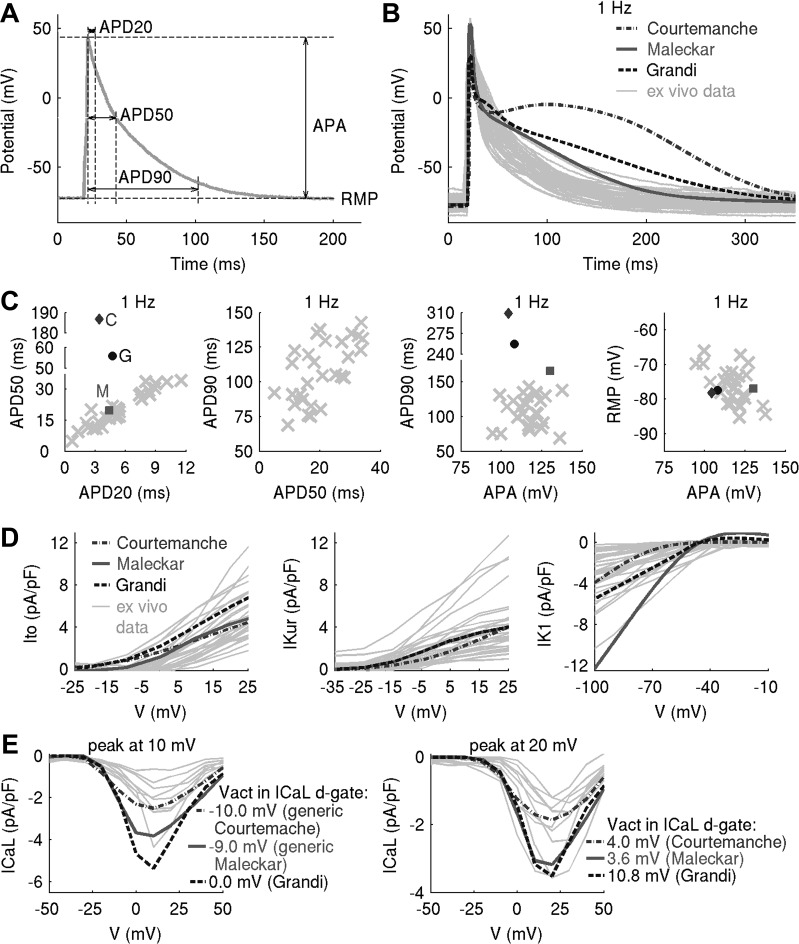
Experimental variability in action potential (AP) and current densities cannot be captured with a single in silico model of human atrial electrophysiology, motivating the use of a population-of-models approach. *A*: AP biomarkers used in this study. APD_20_, APD_50_, and APD_90_, AP duration at 20%, 50%, and 90% repolarization, respectively (in ms); RMP, resting membrane potential (in mV); APA, action potential amplitude (in mV); *V*, voltage. *B*–*E*: outputs of generic Courtemanche, Maleckar, and Grandi models compared with experimental AP traces (*B*), AP biomarkers (*C*), and ionic current densities (*D* and *E*). In *C*, outputs of Courtemanche, Maleckar, and Grandi models are represented with a diamond, square, and circle, with ex vivo data plotted as crosses. *E*: two peaks in L-type Ca^2+^ current (*I*_CaL_) density were observed ex vivo. To capture these, modifications to half-potential of activation (*V*_act_) of the *I*_CaL_ d-gate were introduced to generic models (see *Modifications to the generic models to capture two peaks in I_CaL_ density* in the appendix).

Whole cell current- and voltage-clamp experiments were used to measure APs and *I*_to_, *I*_Kur_, *I*_CaL_, and *I*_K1_ densities in isolated human atrial myocytes. Myocytes were obtained from the right atrial appendage of patients in sinus rhythm, aged 69 ± 10 yr and undergoing coronary artery bypass graft or aortic valve replacement (Table 1). APs were measured in *n* = 29 cells at five pacing frequencies (0.25, 0.5, 1, 2, and 3 Hz). Figure 1*A* shows the AP biomarkers computed in this study, that is, APDs at 20%, 50%, and 90% repolarization (APD_20_, APD_50_, APD_90_, respectively), resting membrane potential (RMP), and AP amplitude (APA). In voltage-clamp experiments, pharmacological inhibition and/or voltage steps were used to isolate the desired components of the total transmembrane current. *I*_Kur_, *I*_to_, *I*_CaL_, and *I*_K1_ densities were measured in *n* = 30, 30, 18, and 35 cells, respectively. Figure 1, *B*–*E*, shows our ex vivo data set at the level of the AP and current densities. A detailed description of experimental protocols is provided in *Experimental Data Set* in the appendix. Investigations were approved by the Research Ethics Committee (reference number: 07/Q1607/38), and investigations were conducted in accordance with the principles of the Declaration of Helsinki. All patients gave informed written consent.

**Table 1.** Clinical and demographic characteristics of patients in sinus rhythm

Characteristic	
Total number of patients	35
Age, yr (mean ± SD)	69 ± 10
Men, *n* (%)	26 (74)
Women, *n* (%)	9 (26)
Surgical procedure	
Coronary artery bypass surgery with or without AVR/MVR	22 (63)
AVR/MVR	13 (37)
Medical history	
Smoker/exsmoker	17 (49)
Hypertension	24 (69)
Diabetes mellitus	5 (14)
Heart failure	3 (9)
Previous myocardial infarction	9 (25)
Chronic obstructive pulmonary disease/asthma	3 (9)
Smoker/exsmoker	99 (56)
Medications	
Anticoagulants	4 (11)
Antiplatelets	23 (66)
β-Blockers	23 (66)
Statins	25 (71)
Ca^2+^ channel blockers	8 (23)
Angiotensin-converting enzyme inhibitors and angiotensin II receptor blocker	21 (60)
Diuretics	14 (40)

Values are total numbers of patients with percentages in parentheses unless indicated otherwise. AVR, aortic valve replacement; MVR, mitral valve replacement.

#### Constructing in silico populations of human atrial electrophysiological models.

Ex vivo variability in AP and ionic current data cannot be captured with a single in silico model of human atrial electrophysiology (Fig. 1, *B*–*E*), motivating the use of a population-of-models approach (4). For this purpose, we select the following three generic in silico models from the literature based on the degree of similarity between model outputs and our ex vivo data (Fig. 1, *B*–*C*): *1*) the Maleckar et al. model (23) yields a triangular AP with APD closest to our experimental data; *2*) the Grandi et al. model (15) produces a triangular AP; however, its APD is longer than in the Maleckar et al. model and lies further away from experiment; and *3*) the AP of the Courtemanche et al. model (7) is characterized by a spike-and-dome morphology with the longest APD out of the three models and is the furthest in AP shape and biomarkers from our ex vivo data.

For simplicity, the models are henceforth referred to by their first authors’ names. Interestingly, despite substantial differences in the three models’ AP properties, the ionic currents thought to be the major determinants of the human atrial AP, i.e., *I*_to_, *I*_K1_, *I*_CaL_, and atrial-specific *I*_Kur_, are not only similar between the models but are also within/close to the experimentally observed ranges of variability in our ex vivo data set (Fig. 1, *D* and *E*).

In our experimental data set, peak *I*_CaL_ density occurred at 10 or 20 mV in *n* = 10 and 8 cells, respectively (Fig. 1*E*). Although positively shifted, this is in agreement with reported variability in voltages of peak *I*_CaL_ in human atrial myocytes (28, 30, 37). To capture this in silico, modifications in *I*_CaL_ gating were introduced in the three generic models. Details are provided in *Generic Models of Human Atrial Electrophysiology* in the appendix.

To capture cell-to-cell variability as seen in our experimental data set, we constructed populations of in silico models sharing the same equations but with different parameter sets and in range with experimentally observed variability (4). For every generic model, a corresponding population of models was constructed. As previously discussed (for a review, see Ref. 25), our underlying hypothesis was that variability in ion channel densities is the main determinant of AP variability in cellular electrophysiology. In all three generic in silico models, maximal conductances/permeabilities of *I*_to_, *I*_Kur_, *I*_CaL_, rapid (*I*_Kr_) and slow (*I*_Ks_) components of the delayed rectifier K^+^ current, *I*_K1_, fast Na^+^ current (*I*_Na_), Na^+^-K^+^ pump current (*I*_NaK_), NCX current (*I*_NCX_), uptake current (*J*_up_), and release current (*J*_rel_) were varied simultaneously over ±100% of their baseline model values. This choice of currents was based on their influence on human atrial AP morphology and Ca^2+^ handling following previous investigations and sensitivity analyses (20, 39). A wide range of parameter values was used to explore a variety of possible ionic scenarios reflecting the heterogeneity in the human population and our ex vivo data (Fig. 1, *D* and *E*). Such values are supported by physiological evidence in both inward and outward atrial currents in human patients in sinus rhythm (8, 48, 49). Because there is no experimental evidence on covariation between maximal conductances of two or more ionic currents (12), model parameters were varied independently; should experimental evidence on parameter covariation become available, it can be incorporated into the methodology. To account for heterogeneity in ex vivo peak *I*_CaL_ density (Fig. 1*E*), two versions of every generic model, corresponding to the distinct *I*_CaL_ peaks, were used to build the corresponding in silico population.

To verify that the resultant in silico populations were independent from sampling methodology, the following two methods for generating parameter sets from a high-dimensional parameter space were used: Latin hypercube sampling (LHS; see Ref. 24) and sequential Monte Carlo (SMC; see Ref. 9). Under LHS, the range of values that every varied parameter can take is subdivided into *N* equally spaced intervals, and *N* parameter sets are generated in such a way that every varied parameter takes a value within the specific interval only one time. We use LHS to create a candidate population of *N* = 30,000 models for every generic model of human atrial electrophysiology. This candidate population is then simulated in conditions closely resembling experiment, and a subset of models with biomarkers within experimentally observed ranges are retained for further analysis. The SMC algorithm searches parameter space until it finds a certain number of parameter sets (here 800) that yield in silico models whose outputs agree with our ex vivo AP biomarkers. To locate those target parameter sets, SMC repeatedly resamples models whose AP biomarkers are furthest away from the desired values and then applies Markov chain Monte Carlo steps to these models to explore the parameter space and ensure that most models in the population remain unique. The jumping distribution used for these Markov chain Monte Carlo steps is a three-component Gaussian mixture fitted to the locations of all models in the parameter space. A detailed description of the SMC method is provided in *SMC Sampling for Experimentally Calibrated Populations of Models* in the appendix.

#### Calibration criteria from ex vivo recordings.

**Fig. 2.**
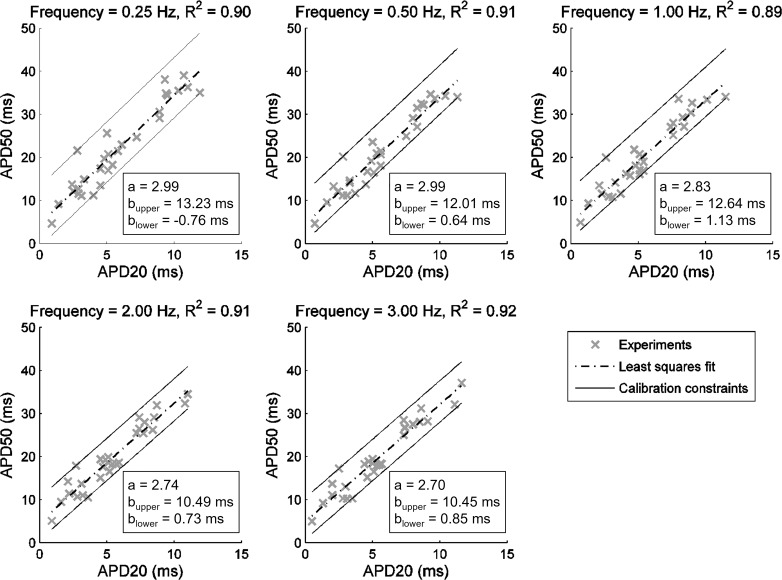
Linear relationship between action potential (AP) duration (APD) at 20% and 50% repolarization (APD_20_ and APD_50_) properties at five pacing frequencies ex vivo. Experimental data are shown as crosses, dashed-dotted lines are the least-squares fits to the data (corresponding *R*^2^ value given at the *top* of each plot), and solid black lines represent constraints corresponding to *a*APD_20_ + *b*_upper_ ≥ APD_50_ ≥ *a*APD_20_ + *b*_lower_ imposed on APD_20_ and APD_50_ properties generated by in silico models. The constant *a* was obtained from the least-squares fit, whereas *b*_lower_ and *b*_upper_ were determined from the values of the outermost experimental data points.

**Fig. 3.**
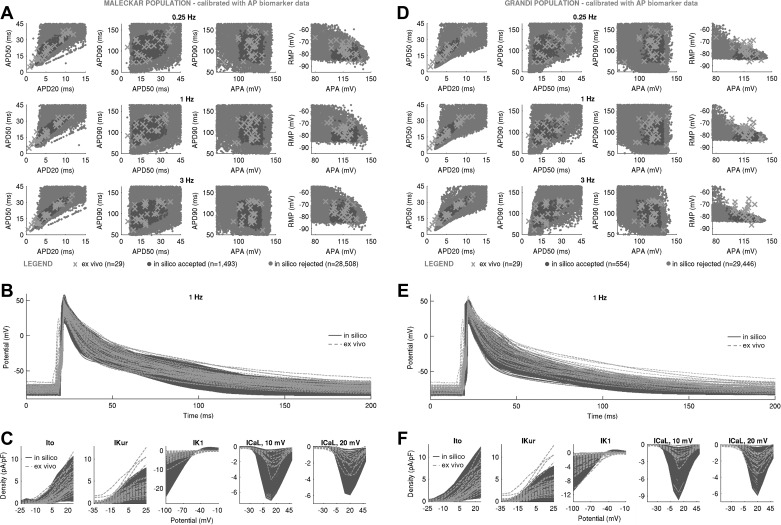
Populations of models with a substantial variability in maximal conductances can yield action potential (AP) and ionic current characteristics overlapping with ex vivo data set even if generic model outputs are far away from experiment. *A***–***C*: in silico AP biomarkers at 0.25, 1, and 3 Hz (*A*), full AP traces (*B*), and ionic current densities of transient outward K^+^ current (*I*_to_), ultrarapid K^+^ current (*I*_Kur_), inward rectifier (*I*_K1_), and L-type Ca^2+^ current (*I*_CaL_) (with peaks at 10 and 20 mV (*C*) in the Maleckar-based population of *n* = 1,493 models following calibration with AP biomarker data compared with ex vivo measurements. *D*–*F*: analogous plots for the Grandi-based in silico population of *n* = 554 models. Both in silico populations were generated with Latin hypercube sampling. Throughout, in silico “accepted” models, meaning those models that fulfill experimental AP calibration constraints, are plotted in dark gray; *A* and *D* additionally show AP biomarkers of in silico “rejected” models in light gray. Ex vivo data are shown as crosses. RMP, resting membrane potential; APA, AP amplitude; APD_20_, APD_50_, and APD_90_, AP duration at 20%, 50%, and 90% repolarization.

Calibration criteria involved retaining the models whose AP and/or ionic properties were in range with ex vivo recordings. Most studies with the use of the experimentally calibrated population-of-models methodology had access to experimental data on AP biomarkers only (25). To investigate the importance of information contained within AP biomarkers versus current densities, we first constrained candidate populations using solely AP data. The experimentally derived constraints imposed on in silico APs consisted of the following: *1*) minimum/maximum values of APD_20_, APD_50_, APD_90_, RMP, APA, and the difference APD_90_ − APD_50_ (triangulation) at all pacing frequencies (Table 2); *2*) constraint of linearity between APD_20_ and APD_50_ observed when ex vivo biomarkers were investigated for covariation (Fig. 2); *3*) APD_90_ rate dependence: the values of APD_90_ at 3 and 0.25 Hz generated by each in silico model were required to fulfill the inequality 1.11 ≥ APD_90_ (3 Hz)/APD_90_ (0.25 Hz) ≥ 0.75 [since the minimum and maximum values of the ratio APD_90_ (3 Hz)/APD_90_ (0.25 Hz) ex vivo were 1.11 and 0.75, respectively]; and *4*) criterion on APD alternans: models whose APD_90_ differed by >5 ms between two final beats were rejected, as in Ref. 52.

**Table 2.** Minimum and maximum bounds of action potential biomarkers, obtained from n = 29 cells ex vivo and used to calibrate the in silico populations of models

Frequency	APD_20_, ms	APD_50_, ms	APD_90_, ms	RMP, mV	APA, mV	Tri_90–50_, ms
0.25 Hz	0.9–11.9 (5.8 ± 3.2)	4.7–39.0 (22.0 ± 10.0)	72.8–154.7 (113.8 ± 23.8)	−86.7 to −66.5 (−76.5 ± 5.4)	79.7–136.5 (116.8 ± 10.9)	56.7–121.4 (91.8 ± 19.4)
0.5 Hz	0.7–11.3 (5.6 ± 2.9)	4.7–34.7 (21.0 ± 8.8)	69.9–147.0 (109.3 ± 22.8)	−85.4 to −67.6 (−76.3 ± 5.1)	86.2–133.1 (117.1 ± 9.8)	54.2–118.9 (88.3 ± 18.8)
1 Hz	0.7–11.5 (5.5 ± 2.8)	4.9–34.1 (20.3 ± 8.3)	68.9–142.8 (107.8 ± 21.9)	−85.2 to −66.1 (−76.2 ± 5.1)	94.6–137.5 (116.8 ± 9.8)	58.3–117.6 (87.5 ± 18.0)
2 Hz	0.9–11.0 (5.5 ± 2.7)	5.1–34.5 (19.8 ± 7.7)	64.4–135.9 (106.1 ± 21.3)	−85.9 to −66.2 (−76.0 ± 4.5)	100.6–130.7 (116.7 ± 8.2)	54.9–113.5 (86.3 ± 17.9)
3 Hz	0.5–11.6 (5.4 ± 2.8)	5.0–37.1 (19.7 ± 8.0)	60.7–131.6 (103.9 ± 21.0)	−87.2 to −65.6 (−76.0 ± 5.2)	92.8–132.1 (115.3 ± 9.7)	51.5–111.4 (84.2 ± 17.7)

Values are ranges with means ± SD in parentheses. APD_20_, APD_50_, and APD_90_, action potential duration at 20%, 50%, and 90% repolarization, respectively; RMP, resting membrane potential; APA, action potential amplitude; Tri_90–50_, triangulation.

In the case of LHS, all experimental constraints on the AP properties were applied at once. Under SMC, the calibration criteria in *points 1* and *2* were applied sequentially for every biomarker (*SMC Sampling for Experimentally Calibrated Populations of Models* in the appendix); to reduce computational burden, *criteria 3* and *4* were applied after the SMC algorithm stopped, i.e., after generating 800 models that fulfilled constraints in *points 1* and *2*.

#### In silico stimulation protocols.

In silico populations were simulated to elicit cellular APs and *I*_to_, *I*_Kur_, *I*_CaL_, and *I*_K1_ densities in conditions resembling experiments as closely as possible (for details, see *Computational stimulation protocols* and *Numerical methods and data analysis* in the appendix). Briefly, we assumed that ionic concentrations in the pipette and extracellular solutions used in experiments corresponded to the intracellular and extracellular concentrations in the models. The intracellular Na^+^ and K^+^ and extracellular Na^+^, K^+^, and Ca^2+^ concentrations were held constant in time and to their experimental equivalents. The temperature was set to 310.15 K in the simulations where the APs and *I*_to_, *I*_Kur_, and *I*_CaL_ current densities were elicited, whereas in the simulations of *I*_K1_ density, the temperature was 295.15 K. To elicit APs, each model was stimulated for 100 beats at every frequency, following the framework designed in Ref. 52. The duration and amplitude of stimulus current were matched to the median experimental values. In voltage-clamp simulations, pharmacological inhibition of specific ion channels was modeled by setting the corresponding maximal conductances to zero.

## RESULTS

### 

#### Populations of models with wide variations in ionic densities can yield APs overlapping with experiments, even if generic models are initially far away from the specific experimental cohort.

Figure 3 shows the AP biomarkers and ionic densities in Maleckar-based and Grandi-based in silico populations produced with the LHS algorithm following calibration with ex vivo AP data. In both populations, substantial variability in maximal conductances was sufficient to generate a wide range of AP characteristics that overlapped with our ex vivo data set, even if the generic model outputs were far from the ex vivo data under consideration (Fig. 1). In the Maleckar-based population, calibration with ex vivo AP biomarkers yielded *n* = 1,493 in silico models with AP properties spanning full ranges of experimentally observed variability at the AP level (Fig. 3, *A* and *B*). Simulated ionic densities largely encompassed ex vivo recordings of inward Ca^2+^ and outward K^+^ currents, with in silico *I*_K1_ and *I*_CaL_ spanning a wider range of values than in our experiments (Fig. 3*C*).

A similar picture could be seen in the Grandi-based population. Of the *N* = 30,000 candidate models generated by LHS, *n* = 554 produced APD and APA properties in range with experimental observations (Fig. 3, *D* and *E*), with simulated RMP biomarkers occupying the bottom half of the experimentally observed range of values. Ionic current densities in the Grandi-based population overlapped with ex vivo recordings, with some models exceeding peak *I*_CaL_ density recorded in experiments (Fig. 3*F*).

**Fig. 4.**
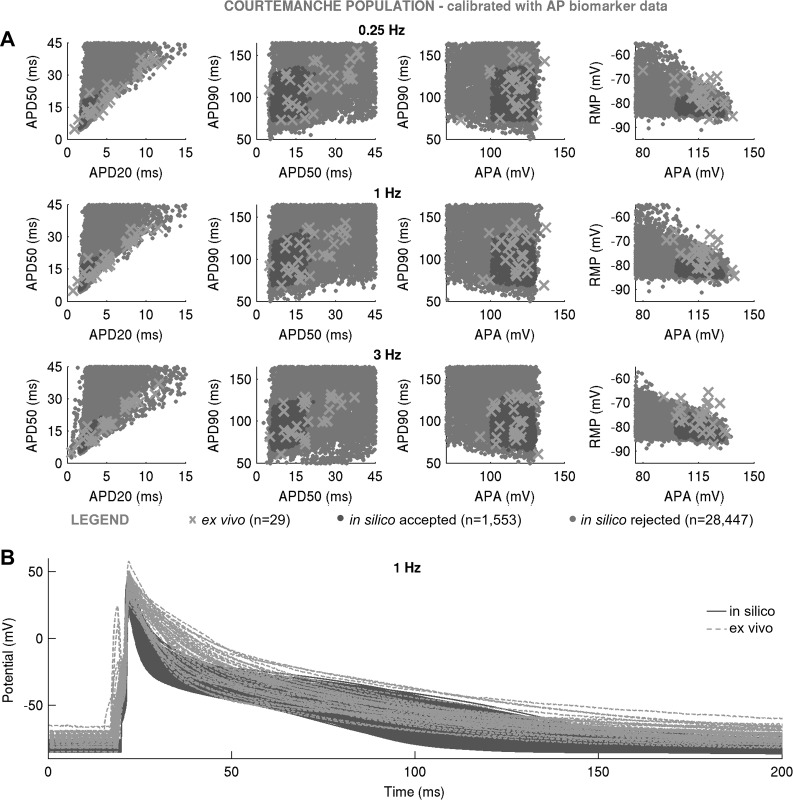
In silico action potential (AP) biomarkers at 0.25, 1, and 3 Hz (*A*) and full AP traces in the Courtemanche-based population of *n* = 1,553 models (*B*) following calibration with AP biomarker data compared with ex vivo measurements. In silico population was generated with Latin hypercube sampling. In silico accepted models, i.e., those models that fulfill experimental AP calibration constraints, are plotted in dark gray; *A* additionally shows AP biomarkers of in silico rejected models in light gray. Ex vivo data are shown as crosses.

**Fig. 5.**
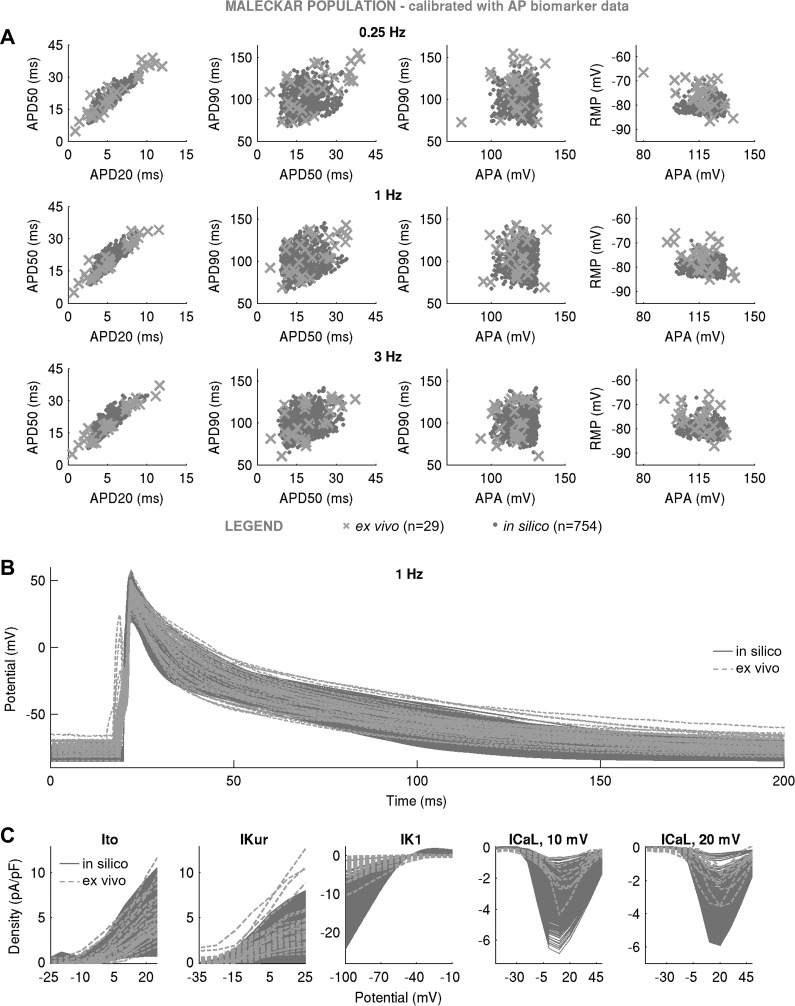
In silico action potential (AP) biomarkers at 0.25, 1, and 3 Hz (*A*), full AP traces (*B*), and ionic current densities (*C*) of transient outward K^+^ current (*I*_to_), atrial-specific ultrarapid K^+^ current (*I*_Kur_), inward rectifier K^+^ current (*I*_K1_), and L-type Ca^2+^ current (*I*_CaL_) (with peaks at 10 and 20 mV) in the Maleckar-based population of *n* = 754 models following calibration with AP biomarker data compared with ex vivo measurements. The in silico population was generated with sequential Monte Carlo. In silico accepted models, i.e., those models that fulfill experimental AP calibration constraints, are plotted in gray dots, whereas ex vivo data are shown as crosses.

**Fig. 6.**
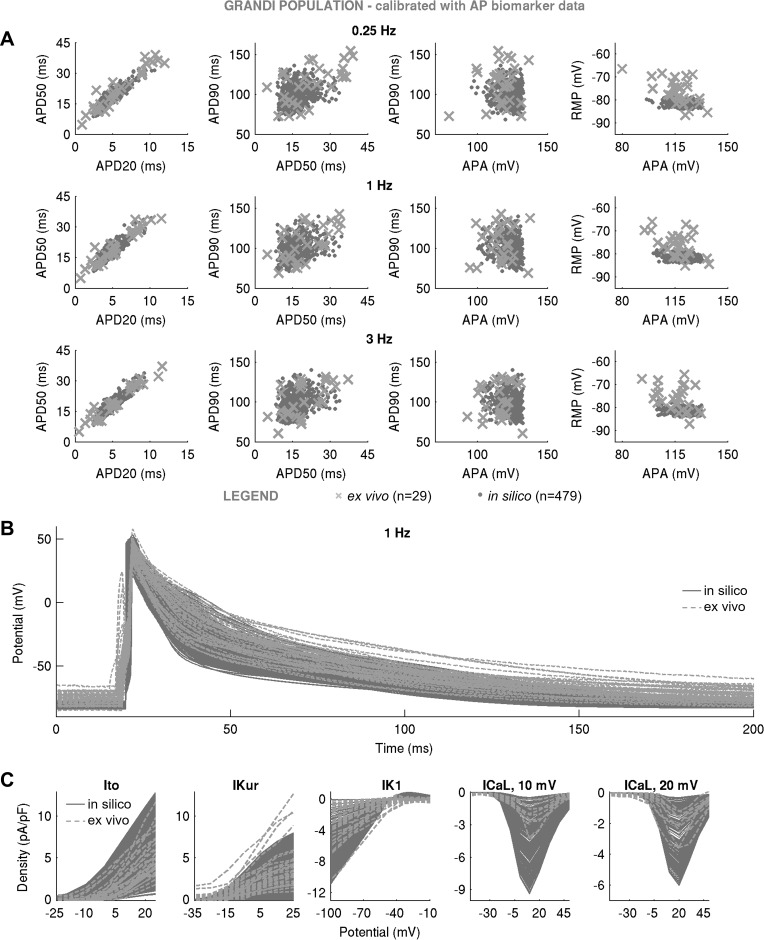
In silico action potential (AP) biomarkers at 0.25, 1, and 3 Hz (*A*), full AP traces (*B*), and ionic current densities (*C*) of transient outward K^+^ current (*I*_to_), atrial-specific ultrarapid K^+^ current (*I*_Kur_), inward rectifier K^+^ current (*I*_K1_), and L-type Ca^2+^ current (*I*_CaL_) (with peaks at 10 and 20 mV) in the Grandi-based population of *n* = 479 models following calibration with AP biomarker data compared with ex vivo measurements. In silico population was generated with sequential Monte Carlo. In silico accepted models, i.e., those models that fulfill experimental AP calibration constraints, are plotted in light gray, whereas ex vivo data are shown as crosses.

**Fig. 7.**
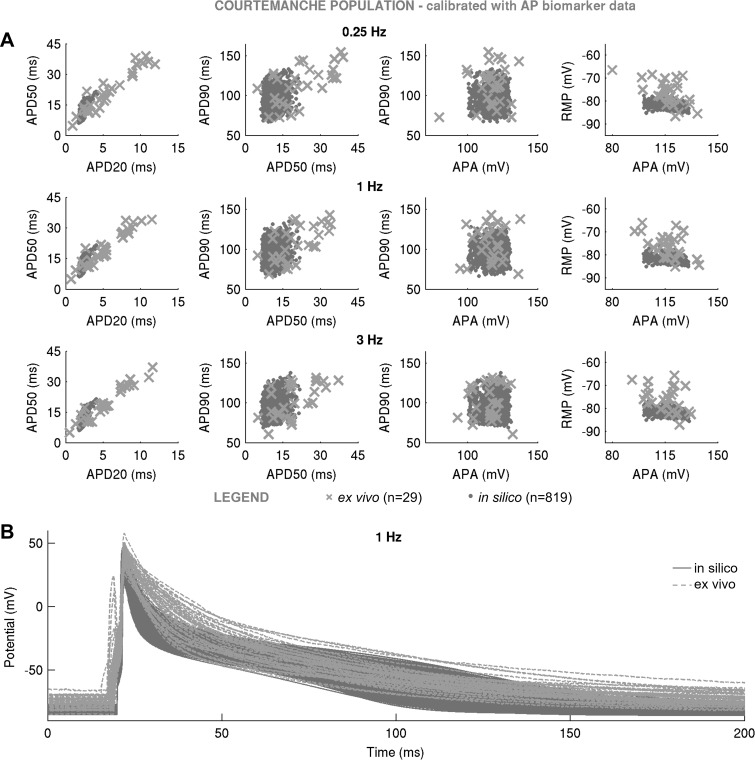
In silico action potential (AP) biomarkers at 0.25, 1, and 3 Hz (*A*) and full AP traces (*B*) in the Courtemanche-based population of *n* = 819 models following calibration with AP biomarker data compared with ex vivo measurements. In silico population was generated with sequential Monte Carlo. In silico accepted models, i.e., those models that fulfill experimental AP calibration constraints, are plotted as light gray, whereas ex vivo data are shown as crosses.

Not all models succeeded in reproducing the variability of the AP recordings. Despite the wide variability in maximal conductances, the Courtemanche-based in silico population of *n* = 1,553 models failed to cover experimental AP ranges, particularly the early repolarization phase (Fig. 4). This is because of the spike-and-dome morphology of the population’s APs, capable of producing APD_20_ and APD_50_ only at the low end of values recorded in our ex vivo data set (Fig. 4), thus failing to yield the long APD_20_ and APD_50_ seen in our experimental data set. The findings of the Courtemanche-based, Maleckar-based, and Grandi-based populations were replicated with the SMC algorithm (Figs. 5–7), highlighting the independence of our results from the specific method used to randomly sample the conductance values in the population. In the remainder of this report, we focus our attention on the Maleckar-based and Grandi-based in silico populations, since they captured experimental ranges of variability in the AP.

#### Calibration with AP data constrains currents affecting upstroke and resting potential, but current redundancy in repolarization allows wide ranges of variability in currents impacting APD.

**Fig. 8.**
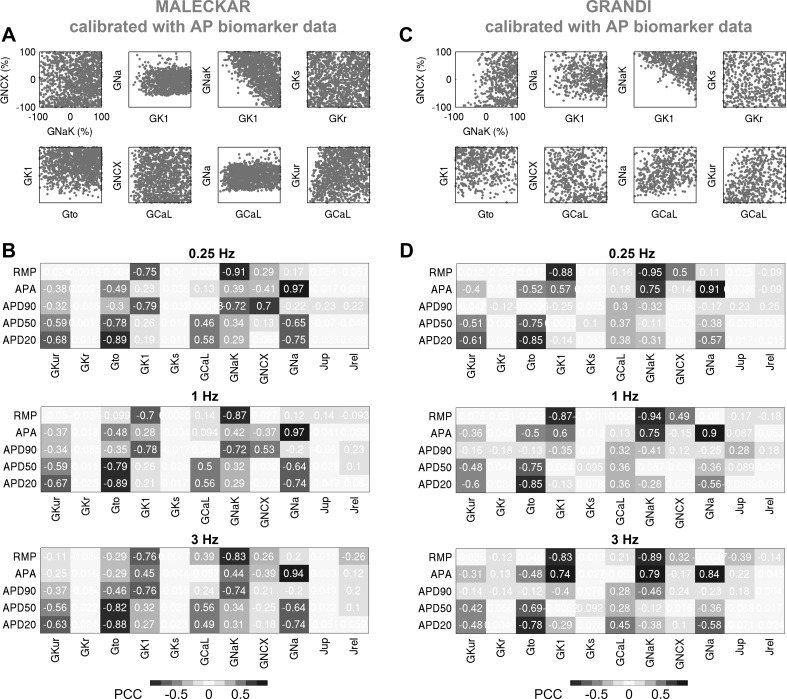
Calibration with action potential (AP) data constrains currents affecting upstroke and resting potential, but current redundancy during repolarization allows wide ranges of variability in currents impacting AP duration (APD). *A* and *B*: selected pairwise plots of maximal conductances (*A*) and partial correlation coefficients (PCCs) between AP biomarkers and maximal conductances (*B*), computed at 0.25, 1, and 3 Hz, for the Maleckar-based population. *C* and *D*: analogous results for the Grandi-based population. In *B* and *D*, positive (+) and negative (−) correlations are highlighted.

Figure 8 shows scatterplots of key maximal conductances, and their correlation with AP biomarkers for the Maleckar-based and Grandi-based populations, calibrated with AP data. In the Maleckar-based population, models with Na^+^ conductance below −59% of baseline are absent from the population because of the critical role of *I*_Na_ in the generation of the AP amplitude (Fig. 8*B*). Models with low Na^+^-K^+^ pump conductance (*G*_NaK_) and low inward rectifier conductance (*G*_K1_) are absent from both AP-calibrated populations because of the critical importance of *I*_NaK_ and *I*_K1_ for RMP (Fig. 8, *B* and *D*). The current conductances regulating cellular repolarization are unconstrained by calibration with AP data because multiple ionic currents regulate APD biomarkers (for instance, APD_20_ is determined by a balance of the following four ionic currents: *I*_to_, *I*_Kur_, *I*_CaL_, and *I*_Na_; Fig. 8, *B* and *D*).

We can now rationalize the impact of AP-level calibration on the simulated current densities shown in Fig. 3, *C* and *F*. It is important to note that the experimental measurements of a specific current density and cellular AP were not obtained simultaneously from the same cell, i.e., our experimental recordings of *I*_to_, *I*_Kur_, *I*_K1_, and *I*_CaL_ do not underpin our experimental APs (furthermore, the cells used for measurements of one current were different from those used for recordings of another current). Figure 3, *C* and *F*, highlights large variability in peak current density observed in experiments and simulations. In both Maleckar-based and Grandi-based populations calibrated with AP-level data, the ranges of ultrarapid K^+^ conductance (*G*_Kur_) and transient outward K^+^ conductance parameters spanned ±100% of their baseline values (Fig. 8, *A* and *C*), translating into simulated *I*_Kur_ and *I*_to_ densities that cover the bulk of our experimental data (Fig. 3, *C* and *F*).

Differences are observed in the ranges of *I*_K1_ density between Maleckar-based and Grandi-based populations. In both populations, high *G*_NaK_ can compensate for low *G*_K1_ and vice versa, as shown in Fig. 8, *A* and *C*. In the Maleckar-based population, some models displayed a peak *I*_K1_ density of up to −24.62 pA/pF (Fig. 3*C*), exceeding the value of −10.33 pA/pF seen in our ex vivo data, whereas the Grandi-based population produced *I*_K1_ density in range with our experimental recordings (Fig. 3*F*). This interpopulation difference is the result of distinct experimental data sets used in the construction of the generic Maleckar and Grandi models. The generic Maleckar model yielded *I*_K1_ density of −12.31 pA/pF (at −100 mV) and was based on previous experimental data (3) where *I*_K1_ density of −9.83 ± 0.96 pA/pF (at −90 mV) was reported. The Grandi model, derived from a model of a human ventricular myocyte, takes into account the sixfold smaller density of *I*_K1_ in atria relative to ventricles (15, 42) and yields a baseline *I*_K1_ density of −5.49 pA/pF, close to the experimentally measured value of −3.5 ± 0.3 (at −120 mV) previously reported (55). The threefold difference in experimental measurements of *I*_K1_ density previously reported (3, 55) provides another illustration of the wide heterogeneity in the human population.

In both in silico populations calibrated with AP data, *I*_CaL_ was not the main determinant of AP, as evidenced by its weak correlation with AP properties (Fig. 8, *B* and *D*). Accordingly, the L-type Ca^2+^ conductance (*G*_CaL_) parameter spanned ±100% of generic model values in both populations (Fig. 8, *A* and *C*). In some of the Maleckar-based and Grandi-based models, *I*_CaL_ density was as high as −7 and −10 pA/pF, both of which exceeded the maximal *I*_CaL_ density of −4.35 pA/pF in our ex vivo recordings (Fig. 3, *C* and *F*). However, peak *I*_CaL_ density ranging from −0.1 to −9 pA/pF (see Fig. 1 in Ref. 8) and from ~0 to −55 pA/pF (see Fig. 3 in Ref. 49) have been reported in patient cohorts in sinus rhythm similar to ours. Therefore, models with larger peak *I*_CaL_ are still representative of the variability observed in sinus rhythm.

#### Further calibration with ionic currents active during repolarization constrains current density but not cellular phenotypic variability in AP.

**Fig. 9.**
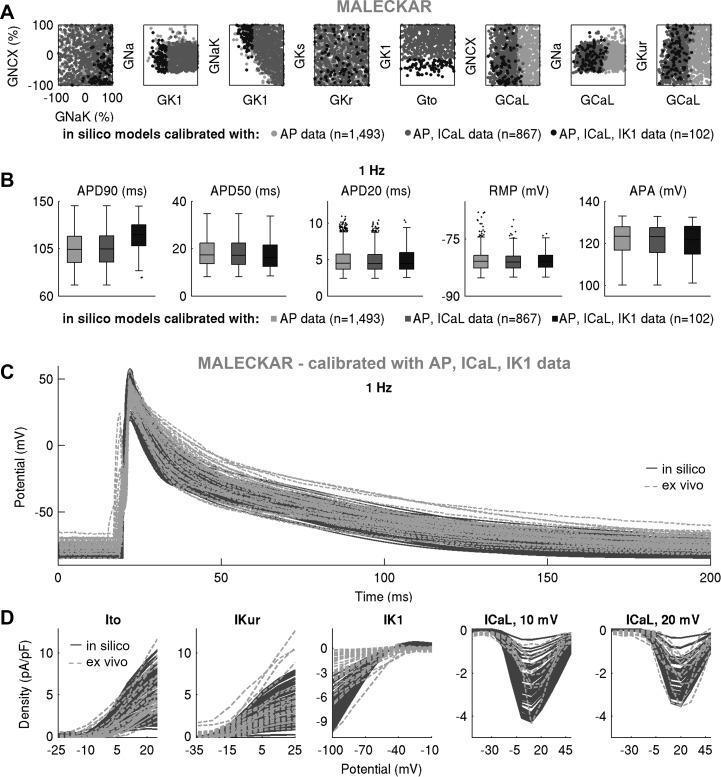
Further calibration with ionic currents active during repolarization constrains current density but not cellular phenotypic variability in action potential (AP). *A* and *B*: pairwise scatter plots of maximal conductances (*A*) and AP biomarkers (*B*) in the populations calibrated with AP, AP + L-type Ca^2+^ current (*I*_CaL_), and AP + *I*_CaL_ + inward rectifier K^+^ current (*I*_K1_) ex vivo data in the Maleckar-based in silico population, plotted in light gray, dark gray, and black, respectively. *C* and *D*: AP traces (*C*) and ionic current densities (*D*) in the Maleckar-based population of *n* = 102 models calibrated with AP and voltage-clamp recordings, shown in dark gray, compared with ex vivo data in light gray.

**Fig. 10.**
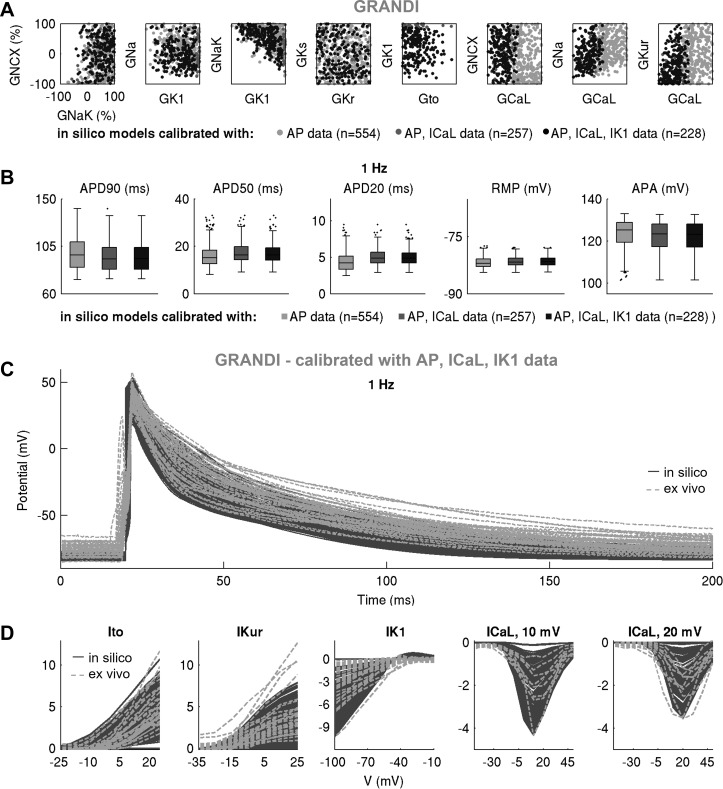
Further calibration with ionic currents active during repolarization constrains current density but not cellular phenotypic variability in action potential (AP). *A* and *B*: pairwise scatterplots of maximal conductances (*A*) and AP biomarkers (*B*) in the populations calibrated with AP, AP + L-type Ca^2+^ current (*I*_CaL_), and AP + *I*_CaL_ + inward rectifier K^+^ current (*I*_K1_) ex vivo data in the Grandi-based in silico population, plotted in light gray, dark gray, and black, respectively. *C* and *D*: AP traces (*C*) and ionic current densities (*D*) in the Maleckar-based population of *n* = 228 models calibrated with AP and voltage-clamp recordings, shown in dark gray, compared with ex vivo data in light gray.

**Fig. 11.**
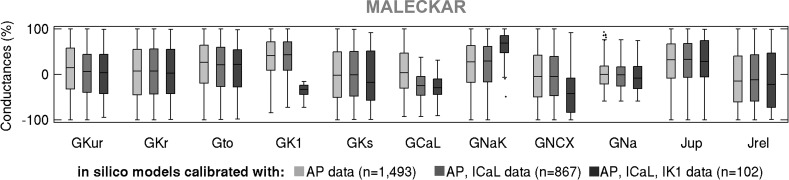
Box plots of maximal current conductances in the Maleckar-based in silico population of models following calibration with AP, AP + *I*_CaL_, and AP + *I*_CaL_ + *I*_K1_ data, shown in light gray, dark gray, and black, respectively. Black dots indicate outliers.

**Fig. 12.**
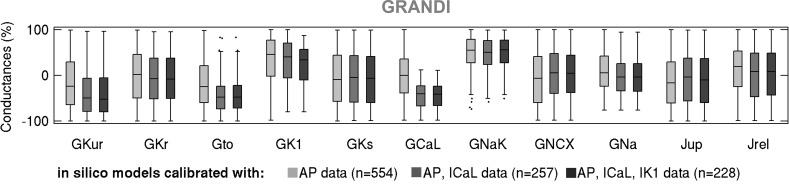
Box plots of maximal current conductances in the Grandi-based in silico population of models following calibration with action potential (AP), AP + L-type Ca^2+^ current (*I*_CaL_), and AP + *I*_CaL_ + inward rectifier K^+^ current (*I*_K1_) data, shown in light gray, dark gray, and black, respectively. Black dots indicate outliers.

We proceeded to further calibrate the two in silico populations using voltage-clamp *I*_CaL_ and *I*_K1_ measurements by retaining models whose peak current densities do not exceed the maximal values in our ex vivo data set. Figures 9 and 10 show the impact of this additional calibration on maximal conductances, AP biomarkers, and peak current densities in the Maleckar-based and Grandi-based populations, respectively. In both populations, additional calibration constraint with *I*_CaL_ density had no effect on maximal conductances other than *G*_CaL_ or on AP biomarkers (Figs. 9*A*, 10*A*, 11, and 12). This is in line with the weak correlation of *G*_CaL_ with AP properties in Fig. 8 and further illustrates the limited role of *I*_CaL_ on cellular phenotypic variability in the AP in our data set.

In contrast, the impact of additional calibration with peak *I*_K1_ density differed between in silico populations. The Maleckar-based population calibrated with AP data contained many models with *I*_K1_ current exceeding the peak value seen in our ex vivo recordings. Thus, additional calibration on *I*_K1_ density substantially reduced the number of viable models and impacted not only the distribution of *G*_K1_ but also of *G*_NaK_ (since those parameters are correlated; Fig. 8*A*). Because of the *I*_K1_ importance in determining APD_90_, few models with short APD_90_ survived this additional calibration step (Fig. 9*B*). In contrast, in the Grandi-based population calibrated with AP-level data, simulated *I*_K1_ density effectively spanned the ex vivo range; hence, the calibration step on peak *I*_K1_ density had little impact on the parameter space or AP biomarkers (Fig. 10). Following the calibration with both AP and voltage-clamp level information, we obtained Maleckar-based and Grandi-based in silico populations with AP and ionic densities in range with our ex vivo data set (Fig. 9, *C* and *D*, and Fig. 10, *C* and *D*).

#### In both in silico populations, Ca^2+^ transient properties are mainly determined by NCX and RyR/SERCA rather than I_CaL_.

**Fig. 13.**
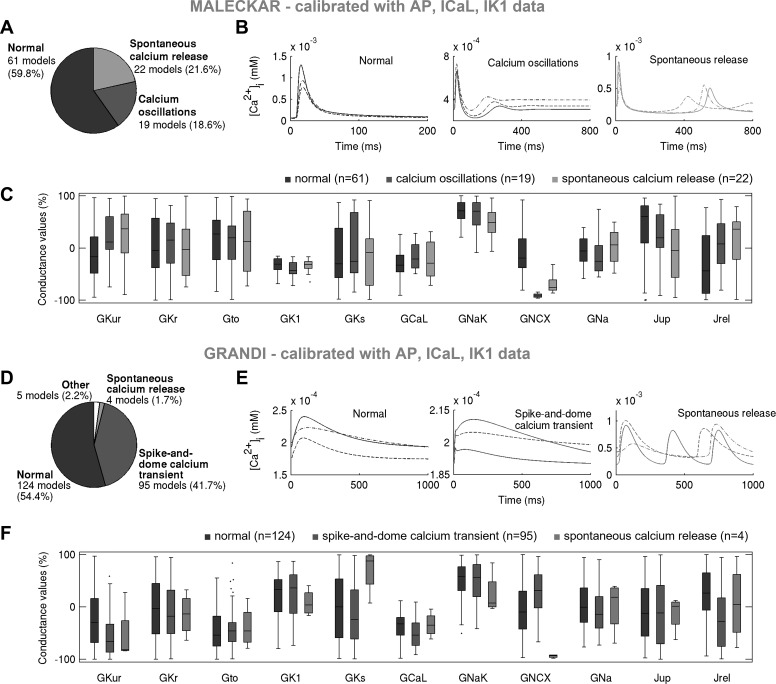
Low Na^+^/Ca^2+^ exchanger current (*I*_NCX_) promotes fluctuations in diastolic Ca^2+^ concentration in both in silico populations, whereas high Na^+^/Ca^2+^ exchanger conductance (*G*_NCX_) and low release current (*J*_rel_) correlate with the presence of a spike-and-dome Ca^2+^ transient (CaT) in the Grandi-based population only. *A*–*C*: proportions of models and examples exhibiting distinct CaT morphologies in the Maleckar population calibrated with action potential (AP) + L-type Ca^2+^ current (*I*_CaL_) + inward rectifier K^+^ current (*I*_K1_) data and parameters underpinning the three CaT phenotypes (*C*). *D*–*F*: showcase analogous results for the Grandi-based population following AP + *I*_CaL_ + *I*_K1_ calibration. In *A*–*C*, morphologically normal CaTs, Ca^2+^ oscillations in diastole, and spontaneous Ca^2+^ release phenotypes are plotted in black, dark gray, and light gray, respectively. In *D*–*F*, morphologically normal CaTs, spike-and-dome CaTs, and spontaneous Ca^2+^ release models are shown in black, dark gray, and light gray, respectively. Ca^2+^ oscillations and spontaneous Ca^2+^ release are referred to as fluctuations in diastolic Ca^2+^.

**Fig. 14.**
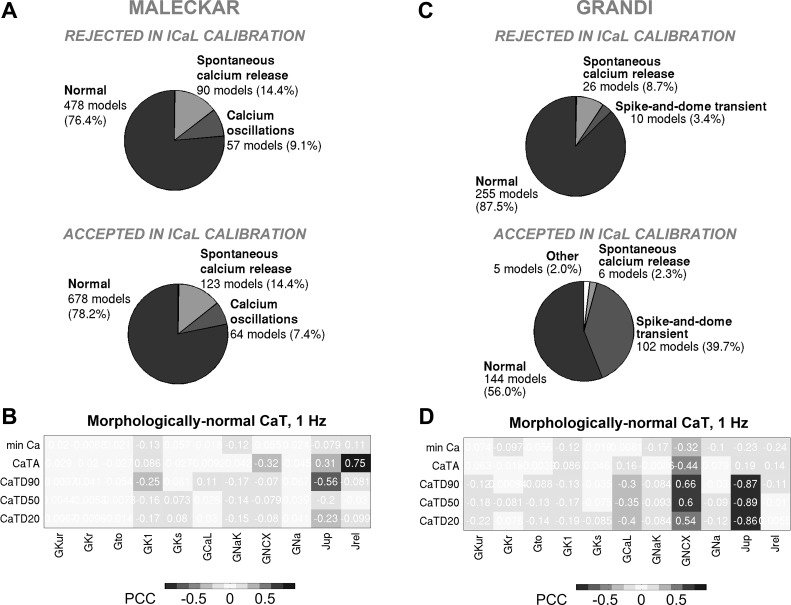
Calibration with L-type Ca^2+^ current (*I*_CaL_) density does not impact proportions of models with distinct Ca^2+^ transient (CaT) phenotypes in the Maleckar population but promotes spike-and-dome CaT morphology in Grandi-based models; key determinants of morphologically normal CaTs are Na^+^/Ca^2+^ exchanger current (*I*_NCX_) and sarcoplasmic reticulum release/uptake currents. *A* and *B*: prevalence of distinct CaT morphologies in the models rejected (*top*) and accepted (*bottom*) in the *I*_CaL_ calibration step (*A*) and partial correlation coefficients (PCCs) between maximal conductances and CaT biomarkers in the Maleckar population following *I*_CaL_ calibration (*B*). *C* and *D*: analogous plots for the Grandi-based population. min Ca, diastolic Ca^2+^ concentration; CaTA, CaT amplitude; CaTDxx, CaT duration at xx% relaxation. Positive (+) and negative (−) correlations are highlighted.

To further explore variability in human atrial models, we analyzed Ca^2+^ transient (CaT) properties in both in silico populations. Figure 13 shows the main CaT types in both populations calibrated with experimental AP and voltage-clamp information, whereas Fig. 14 shows the impact of calibration with peak *I*_CaL_ density and highlights the ionic determinants of CaTs. Given that we considered ±100% variation in many ionic densities, including in the sarcoplasmic reticulum release and uptake currents (*J*_rel_ and *J*_up_; Figs. 11 and 12), a wide variability in the biomarkers and morphology of the simulated CaTs can be expected.

The models within the Maleckar-based population exhibited the following three CaT types (Fig. 13, *A* and *B*): the morphologically normal transient, seen in 60% of the models; Ca^2+^ oscillations in diastole, observed in 21% of the models and previously reported in ex vivo multicellular tissue preparations (33); and spontaneous Ca^2+^ release from the sarcoplasmic reticulum during diastole, observed in 19% of the models. The proportions of models with the three CaT types did not change substantially with *I*_CaL_ calibration (Fig. 14*A*), illustrating that the CaT phenotypes in the Maleckar-based population are independent of *I*_CaL_ density.

The Grandi-based population calibrated with AP and voltage-clamp level data contained three main CaT types (Fig. 13, *D* and *E*) as follows: the morphologically normal transient, exhibited by 55% of the models; the spike-and-dome CaT characterized by the presence of two peaks in CaT curve during systole and seen in 42% of the models; and spontaneous Ca^2+^ release in diastole, observed in 2% of the models. The proportion of models with the spike-and-dome CaT morphology increased significantly with *I*_CaL_ calibration (Fig. 14*C*), suggesting that low *I*_CaL_ may promote this CaT phenotype in Grandi-based models.

**Fig. 15.**
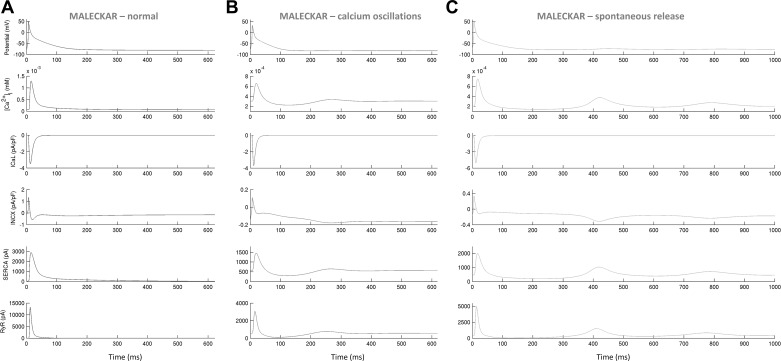
Ionic processes underpinning morphologically normal Ca^2+^ transients (*A*), oscillations in diastolic Ca^2+^ (*B*), and spontaneous Ca^2+^ release during diastole (*C*) in selected Maleckar-based in silico models. In *B* and *C*, fluctuations in diastolic Ca^2+^ are preceded by Ca^2+^ release from the sarcoplasim reticulum [ryanodine receptor (RyR), *bottom*].

**Fig. 16.**
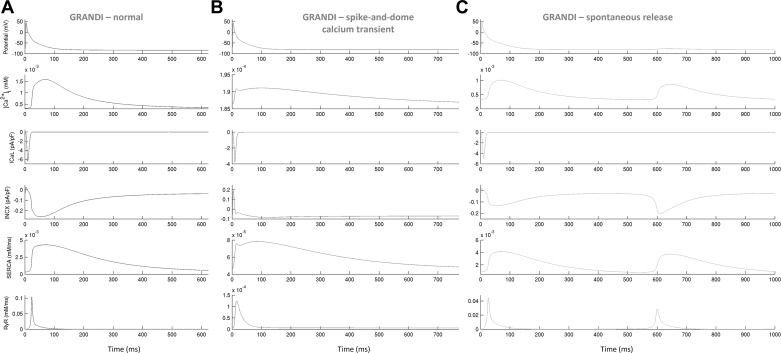
Ionic processes underpinning the morphologically normal Ca^2+^ transient (CaT; *A*), spike-and-dome CaT (*B*), and spontaneous Ca^2+^ release (*C*) during diastole in selected Grandi-based in silico models. In *B*, the spike in CaT coincides with a prominent notch in the forward-mode Na^+^/Ca^2+^ exchanger current (*I*_NCX_); weak Ca^2+^ release from the sarcoplasim reticulum can be seen [ryanodine receptor (RyR), *bottom*]. In *C*, fluctuations in diastolic Ca^2+^ are preceded by Ca^2+^ release from the sarcoplasim reticulum.

In both in silico populations, fluctuations in diastolic Ca^2+^ (inclusive of Ca^2+^ oscillations in diastole and spontaneous Ca^2+^ release phenotypes) were observed in models with low NCX conductance (*G*_NCX_; Fig. 13, *C* and *F*). Additionally, plots of ionic currents involved in CaT generation implicated a release from the sarcoplasmic reticulum during diastole (Figs. 15 and 16). In a study on guinea pig hearts ex vivo, Plummer et al. (33) reported multicellular diastolic Ca^2+^ fluctuations, observing that RyR inhibition abolished those fluctuations, whereas increasing RyR open probability exacerbated them. Consistent with this, a reduction of *J*_rel_ by 90% suppressed most diastolic Ca^2+^ fluctuations, whereas increasing *J*_rel_ by 100% had the opposite effect in both in silico populations (Tables 3 and 4). Separately, increasing *G*_NCX_ to normal levels abolished most Ca^2+^ fluctuations in diastole in both populations (Tables 3 and 4).

**Table 3.** Number of models exhibiting fluctuations in diastolic Ca^2+^ in the Maleckar-based in silico population calibrated with AP + I_CaL_ + I_K1_ data in the absence and presence of interventions on J_rel_ and I_NCX_

	Following AP + *I*_CaL_ + *I*_K1_ calibration	90% Block *J*_rel_	100% Increase *J*_rel_	Increase *I*_NCX_ to Normal Levels
Number of models with Ca^2+^ oscillations	22	0	22; more peaks and larger amplitude of oscillations	3
Number of models with spontaneous Ca^2+^ release	19	0	19; more peaks and larger amplitude of oscillations	0
Number of models with fluctuations in diastolic Ca^2+^	43	0	43	3

AP, action potential; *I*_CaL_, L-type Ca^2+^ current; *I*_K1_, inward rectifier K^+^ current; *J*_rel_, release current; *I*_NCX_, Na^+^/Ca^2+^ exchanger current. 90% block of *J*_rel_ abolishes fluctuations in all models; 100% increase in *J*_rel_ exacerbates diastolic Ca^2+^ fluctuations (more peaks in diastolic Ca^2+^, larger amplitude of Ca^2+^ fluctuations). Increasing *I*_NCX_ to normal levels reduces the number of models with diastolic Ca^2+^ fluctuations from 43 to 3.

**Table 4.** Number of models exhibiting fluctuations in diastolic Ca^2+^ in the Grandi-based in silico population calibrated with AP + I_CaL_ + I_K1_ data in the absence and presence of interventions on J_rel_ and I_NCX_.

	Following AP + *I*_CaL_ + *I*_K1_ Calibration	90% Block *J*_rel_	100% Increase *J*_rel_	Increase *I*_NCX_ to Normal Levels
Number of models with spontaneous Ca^2+^ release	4	1	4; more peaks and smaller amplitude of oscillations	0

AP, action potential; *I*_CaL_, L-type Ca^2+^ current; *I*_K1_, inward rectifier K^+^ current; *J*_rel_, release current; *I*_NCX_, Na^+^/Ca^2+^ exchanger current. 90% block of *J*_rel_ abolishes fluctuations in 3 of 4 models; 100% increase in *J*_rel_ exacerbates diastolic Ca^2+^ fluctuations (more peaks in diastolic Ca^2+^, smaller amplitude of Ca^2+^ fluctuations). Increasing *I*_NCX_ to normal levels abolishes fluctuations in all models.

An interesting feature of some models within the Grandi-based population is the spike-and-dome CaT morphology, coinciding with strong extrusion of Ca^2+^ from the myocyte (high *G*_NCX_; Fig. 13*F*) and a weak injection of Ca^2+^ in the cytosol [low *J*_rel_ (Fig. 13*F*) and/or low *I*_CaL_ (Fig. 14*C*)]. The spike in the CaT during systole coincides with a prominent “notch” in forward-mode *I*_NCX_ (Fig. 16). Accordingly, 50% *G*_NCX_ block to diminish strong extrusion of Ca^2+^ or a 50% *J*_rel_/*I*_CaL_ increase for a stronger injection of Ca^2+^ in the cytosol (to compensate for the effect of *I*_NCX_ notch), all substantially reduced the number of models with this CaT phenotype (Table 5). In summary, in the Maleckar-based population, the biomarkers of morphologically normal CaTs are mainly determined by sarcoplasmic reticulum release and uptake currents (*J*_rel_ and *J*_up_; Fig. 14*B*), whereas *G*_NCX_ and *J*_rel_ control the appearance of diastolic Ca^2+^ fluctuations. In the Grandi-based population, *G*_NCX_ and *J*_up_ are responsible for the properties of morphologically normal CaTs (Fig. 14*D*); additionally, *G*_NCX_ and *J*_rel_ control both the diastolic Ca^2+^ fluctuations and spike-and-dome CaT phenotypes, with the latter also modulated by *G*_CaL_.

**Table 5.** Number of models with a spike-and-dome Ca^2+^ transient in the Grandi-based in silico population calibrated with AP + I_CaL_ + I_K1_ data in the absence and presence of interventions on I_NCX_, J_rel_, and I_CaL_

	Following AP + *I*_CaL_ + *I*_K1_ Calibration	50% Block *I*_NCX_	50% Increase *J*_rel_	50% Increase *I*_CaL_
Number of models with a spike-and-dome Ca^2+^ transient	95	43	67	47
Number of models that lost spike-and-dome CaT morphology (recovering a morphologically normal Ca^2+^ transient) following an intervention, %	NA	55%	30%	51%

AP, action potential; *I*_CaL_, L-type Ca^2+^ current; *I*_K1_, inward rectifier K^+^ current; *J*_rel_, release current; *I*_NCX_, Na^+^/Ca^2+^ exchanger current; NA, not applicable. 50% block of *I*_NCX_ abolishes the spike-and-dome Ca^2+^ transient morphology in 55% of the models, whereas 50% increase in *J*_rel_ and *I*_CaL_ abolish the spike-and-dome morphology in 30% and 51% of the models.

## DISCUSSION

In this report, we describe a combined experimental and computational investigation to deepen our quantitative understanding of cellular and ionic variability in human right atrial myocytes. We developed experimentally calibrated populations of in silico models based on an ex vivo data set of AP and current density recordings obtained in right atrial myocytes from patients undergoing coronary revascularization or aortic valve replacement. Our main findings are as follows: *1*) variability in human atrial APs and ionic currents is wide, with large differences observed *a*) within and between experimental data sets from different patient cohorts (8, 39, 49, 50) and *b*) between APs/ionic densities in experimental recordings versus generic in silico models; *2*) ex vivo cell-to-cell variability in peak current densities from human atrial cardiomyocytes is substantial and can translate to a  ±100% variation in maximal conductances in populations of in silico models of human electrophysiology; *3*) populations of models with a substantial variability in maximal conductances can yield AP characteristics overlapping with ex vivo data set even if outputs of in silico generic models are far away from experiment; *4*) calibration with AP data constrains currents affecting upstroke and resting potential, but current redundancy in repolarization allows wide ranges of variability in currents impacting APD; *5*) additional calibration with ionic currents active during repolarization constrains current density but not cellular phenotypic variability in AP; and *6*) experimentally calibrated populations of atrial electrophysiological models exhibit three Ca^2+^ transient phenotypes, mainly determined by NCX and RyR/SERCA magnitudes rather than *I*_CaL_. The appearance of Ca^2+^ fluctuations in diastole can be caused by low *I*_NCX_, in addition to the previously reported effect of elevated sarcoplasmic release (33).

Our experimental data yielded relatively short APD biomarkers (e.g., APD_90_ ranged between 69 and 143 ms at 1 Hz; Table 2) and APs with a triangular morphology. These data are consistent with findings reported by other groups in atrial myocytes from patients in sinus rhythm (41, 45, 55, 56). Other studies have reported APD_90_ values between 190 and 440 ms at 1 Hz in atrial trabecular preparations from patients in sinus rhythm (39), with both triangular and spike-and-dome AP shapes observed. Methodological aspects (e.g., solution composition, temperature, cellular vs. multicellular recordings) and differences in patient cohorts (e.g., mitral valve vs. coronary revasculization patients) are likely to underlie these discrepancies. Such differences also extend to generic in silico models, depending on the data on which they were based. Given the specific electrophysiological characteristics of our cohort, our results might therefore be only generalizable in the case of short triangular APs in sinus rhythm, as also reported by others (41, 45, 55, 56).

At the level of current densities, we demonstrated that variability in cellular ionic profiles ex vivo is substantial and can translate to a  ±100% variation in maximal conductances in the populations of in silico models. The wide range of variability supports the hypothesis that biological systems are generally “sloppy” (17), i.e., tolerant to significant variations of many parameter combinations. We investigated the ability of Courtemanche, Maleckar, and Grandi human single cell models to capture the variability observed in our ex vivo data set. All three generic models yielded in silico populations that spanned the ranges of ex vivo variability in APD_90_. However, because of the spike-and-dome morphology of its APs, the Courtemanche population was limited in producing models with long APD_20_ and APD_50_ as seen in the experimental data set and captured successfully by Maleckar- and Grandi-based populations. Despite the wide range of variability in maximal conductances, the resting potential spanned the bottom one-half of experimentally measured values in all in silico populations. Aside from *I*_K1_ and *I*_NaK_, RMP is critically determined by the concentrations of ionic species on either side of the cell membrane (predominantly K^+^). Introducing a likely experimental variability in K^+^ concentrations could produce models with higher RMP, as previously shown (26, 50). Additional sources of variability could also include cell-to-cell differences in cell volume and membrane capacitance values, as previously shown (34).

The experimental data set used in this work was particularly restrictive in terms of the short APs observed in the investigated cohort. This was in fact reflected when the in silico populations were calibrated against AP biomarkers, with a rate of model pruning significantly higher than in previous studies (4, 20). Our present results further demonstrate that calibration against AP biomarkers can tightly restrict variability in ion channel densities when these strongly correlate with specific AP biomarkers (such as upstroke and resting potential). Contrastingly, redundancy in plateau and repolarization currents allows wider ranges of variability, and additional ionic density measurements may prove useful in further constraining some of these currents (as with *I*_CaL_ here in both Maleckar- and Grandi-based populations). Although of relevance for previous studies on cell-to-cell variability in which only AP recordings were available and similar ranges of variation in maximal conductances were explored, it is important to recall that those studies aimed to investigate larger, more heterogeneous patient cohorts (20, 39). Given that the mean current density of plateau and repolarization currents has been shown to vary more than fourfold in sinus rhythm in humans (8, 49), larger *I*_CaL_ than in our data may exist at the larger population scale.

A limitation of our experimental data set is that cellular APs and current densities were obtained in different cells. Nevertheless, cells were randomly used from all patients for both AP and ionic current measurements. This should prevent bias and provide a robust rationale for combining both types of measurements. It should be noted that obtaining all measurements in the same cell is not practical, since cells are unlikely to survive exposure to both voltage- and current-clamp configurations, different electrode and bath solutions, and multiple pharmacological interventions and washout periods. It is, however, important to stress that current densities are dynamically changing quantities in vivo, and experimental investigations ex vivo offer valuable but limited snapshots. Considering these complexities, the goal of the experimentally calibrated populations of models is indeed to explore many ionic profiles yielding viable APs by generating larger pools of cellular models than the number of cells that is experimentally feasible to investigate, to overcome some of the experimental limitations.

Further investigations using the resulting in silico populations were conducted to analyze their underpinning Ca^2+^ transients and, in particular, to investigate mechanisms of diastolic Ca^2+^ fluctuations. These were mainly determined by NCX and RyR/SERCA magnitudes rather than *I*_CaL_. Fluctuations were more frequent in the Maleckar-based than Grandi-based population, in some cases leading to delayed afterdepolarizations, consistent with previous findings (6). Importantly, fluctuations in diastolic Ca^2+^ correlated with low NCX expression in both populations, indicating model independence. An implication is that NCX regulates the appearance of diastolic Ca^2+^ disturbances in human atrial cardiomyocytes, in addition to previously reported effects of RyRs (33).

In summary, our study provides further experimental and computational evidence on the large variability in ionic densities and AP recordings in human atrial myocytes and their potential implications in explaining cellular phenotypic variability in transmembrane voltage and Ca^2+^ transient dynamics. Integration between experiments and simulations extends the existing knowledge on the ionic and subcellular mechanisms underlying variability in the human atrial AP and their possible consequences in intracellular Ca^2+^ dysregulation.

## GRANTS

B. Casadei and X. Liu are supported by the British Heart Foundation (BHF; https://www.bhf.org.uk/) through a program grant to B. Casadei (Grant RG/11/15/29375) and the CATCH ME project of the European Union’s Horizon 2020 research and innovation program (Grant 633196). B. Rodriguez is supported by a Wellcome Trust (https://wellcome.ac.uk/) Senior Research Fellowship (100246/Z/12/Z) in Basic Biomedical Science. A. Bueno-Orovio is funded by a BHF Intermediate Basic Science Research Fellowship (FS/17/22/32644). B. Rodriguez and A. Bueno-Orovio also acknowledge additional support from an Impact for Infrastructure Award (NC/P001076/1) of the National Centre for the Replacement, Refinement & Reduction of Animals in Research (https://www.nc3rs.org.uk/). A. Muszkiewicz holds the Engineering and Physical Sciences Research Council (EPSRC) Doctoral Prize and has been funded by an EPSRC scholarship from the Systems Biology Doctoral Training Centre of the University of Oxford. B. A. J. Lawson and K. Burrage are funded by the Australian Research Council (www.arc.gov.au/) through its Centre of Excellence for Mathematical and Statistical Frontiers (CE140100049).

## DISCLOSURES

No conflicts of interest, financial or otherwise, are declared by the authors.

## AUTHOR CONTRIBUTIONS

A.M., X.L., A.B.-O., K.B., B.C., and B.R. conceived and designed research; A.M., X.L., and B.A.L. performed experiments; A.M., X.L., and B.A.L. analyzed data; A.M., X.L., A.B.-O., K.B., B.C., and B.R. interpreted results of experiments; A.M. prepared figures; A.M., X.L., A.B.-O., B.A.L., K.B., B.C., and B.R. drafted manuscript; A.M., X.L., A.B.-O., B.A.L., K.B., B.C., and B.R. edited and revised manuscript; A.M., X.L., A.B.-O., B.A.L., K.B., B.C., and B.R. approved final version of manuscript.

## APPENDIX

### Experimental Data Set

Human atrial myocytes were isolated from the right atrial appendages of patients in sinus rhythm undergoing coronary artery bypass graft/aortic valve replacement/mitral valve replacement in Oxford, UK, as previously described (36). Table 1 shows detailed information on the patient cohort used to perform whole cell voltage and current-clamp experiments in this study.

#### AP measurements.

Patch-clamp experiments were performed with the pipette solution containing (in mM) 130 potassium aspartate, 10 HEPES, 5 Na_2_ATP, 2 MgCl_2_, 5 CaCl_2_, and 11 EGTA (pH adjusted to 7.2 using KOH), whereas the extracellular solution contained (in mM) 140 NaCl, 5.4 KCl, 1.8 CaCl_2_, 1.2 MgCl_2_, 10 glucose, and 5 HEPES (pH adjusted to 7.4 using NaOH). A preexperimental protocol was run to determine the stimulus intensity: APs were recorded in response to 0.25- to 1.5-nA depolarizing current pulses lasting 4 ms with an interval of 1 s using an Axopatch 200 B amplifier in current-clamp mode. The stimulus intensity could be 1.5 times the diastolic threshold. APs from a total of *n* = 29 cells from *N* = 8 patients were measured at five stimulation frequencies (0.25, 0.5, 1, 2, and 3 Hz). Every cell was stimulated a minimum of 25 times; the recorded APs were subsequently averaged to yield a single mean AP trace used to determine the AP properties for each cell. For each cellular AP, RMP, APA, APD_20_, APD_50_, and APD_90_ were quantified.

#### Voltage-clamp data.

Whole cell voltage-clamp experiments were used to determine *I*_to_, *I*_Kur_, *I*_K1_, and *I*_CaL_, as previously detailed in Ref. 36.

To measure *I*_to_, outward currents were evoked by steps in the range of −70 to +50 mV for 700 ms from a holding potential of −80 mV (sampling rate: 5 kHz); a prestep of 200 ms to −40 mV was used to inactivate Na^+^ current. *I*_to_ was measured as the steady-state current subtracted from peak outward current at the start of the depolarizing step and normalized to membrane capacitance value. Steady-state currents were measured at the end of the depolarizing steps. *I*_to_ was measured in *n* = 30 cells from *N* = 7 patients.

To measure *I*_Kur_, outward currents were evoked by steps in the range of −35 to +45 mV for 500 ms from a holding potential of −80 mV; a prestep of 200 ms to −35 mV was used to inactivate Na^+^ current. Sustained current (*I*_sus_) was measured as the amplitude of the current remaining at the end of the pulse relative to the zero current level at the end of the prestep to −35 mV. *4*-*Aminopyridine* (4-AP; 50 μmol/l)-sensitive *I*_Kur_ was obtained by subtracting *I*_sus_ from the remaining current after 4-AP inhibition (53). *I*_Kur_ was measured in *n* = 30 cells from *N* = 7 patients.

In voltage-clamp experiments measuring *I*_Kur_ and *I*_to_, the pipette solution contained (in mM) 120 potassium aspartate, 20 KCl, 5 Na_2_ATP, 2 MgCl_2_, 5 EGTA, and 10 HEPES (pH adjusted to 7.2 using KOH; pCa 7.0). The extracellular solution contained (in mM) 140 NaCl, 5.4 KCl, 1.8 CaCl_2_, 1.2 MgCl_2_, 10 glucose, 5 HEPES, and 0.5 CdCl_2_ (pH adjusted to 7.4 using NaOH). Cd^2+^ was used in experiments to block L-type Ca^2+^ channels. However, Cd^2+^ are known to shift the steady-state activation and inactivation of K^+^ repolarization currents to more positive potentials by ∼20 mV (46). We account for this by shifting experimental current-voltage curves of K^+^ currents by −20 mV.

*I*_K1_ was obtained by digital subtraction of current traces, recorded with a ramp pulse from −100 to +40 mV, in the presence or absence of 1 mmol/l BaCl_2_ (53), as previously described (51). The extracellular solution contained (in mM) 120 NaCl, 20 KCl, 1 MgCl_2_, 1.8 CaCl_2_, 10 glucose, and 10 HEPES (pH 7.4). High extracellular K^+^ was used to shift the reversal potential to more positive values to obtain larger inward currents (51). The pipette solution contained (in mM) 80 potassium aspartate, 8 NaCl, 40 KCl, 2 CaCl_2_, 5 Mg-ATP, 2 EGTA, 0.1 GTP-Tris, and 10 HEPES (pH 7.4). To measure *I*_K1_, *n* = 36 cells from *N* = 7 patients were used.

*I*_CaL_ was elicited with step depolarization protocols using test potentials in the range of −50 to +50 mV in 10-mV increments with a holding potential at −40 mV (43). Recordings were begun and finished after the Ca^2+^ current amplitudes and access resistance had stabilized. *I*_CaL_ was measured as the difference between the current at the end of 250-ms test pulse and the peak inward current. *I*_CaL_ was measured in *n* = 18 cells from *N* = 6 patients. The cells could be split into two groups based on the value of the transmembrane voltage at which the peak inward current was detected, since peak *I*_CaL_ was registered at 10 and 20 mV in 8 and 10 cells, respectively. Pipette solution contained (in mM) 120 Cs-Aspartate, 10 TEA-Cl, 5 MgATP, 2 MgCl_2_, 1 CaCl_2_, 11 EGTA, and 10 HEPES (pH 7.2 with CsOH). The extracellular solution contained (in mM) 140 TEA-Cl, 5.4 CsCl, 1.2 MgCl_2_, 5 HEPES, 11 glucose, and 1.8 CaCl_2_ (pH 7.4 with CsOH).

### Generic Models of Human Atrial Electrophysiology

Three recent models of human atrial electrophysiology by Courtemanche et al. (7), Maleckar et al. (23), and Grandi et al. (15) were used as a basis for the experimentally calibrated atrial cell populations. Throughout the report, we refer to these models by their first authors’ names for simplicity.

In brief, the three models include the major transmembrane currents responsible for AP generation such as *I*_Na_, *I*_Kur_, *I*_to_, *I*_Kr_, *I*_Ks_, *I*_CaL_, *I*_K1_, *I*_NaK_, and *I*_NCX_. The models also incorporate a description of intracellular Ca^2+^ handling that includes the uptake and release currents *J*_up_ (SERCA) and *J*_rel_ (RyR) of the sarcoplasmic reticulum as well as K^+^, Na^+^ and Ca^2+^ homeostasis regulating intracellular ionic concentrations.

However, the three models differ in the mathematical formulations of most of the transmembrane currents, the intracellular Ca^2+^ handling subsystem, and the number of model compartments. The Maleckar model is based on an earlier model of human atrial electrophysiology published by Nygren et al. (28) but includes an updated description of *I*_Kur_ and *I*_to_ repolarization currents. The Grandi model of the human atrial myocyte was derived from an earlier model of ventricular electrophysiology from the same group (16), with the mathematical formulations of *I*_Kur_ and *I*_to_ currents based on the Maleckar model. The Grandi model additionally includes a description of a plateau K^+^ current (*I*_Kp_) and Ca^2+^-activated Cl^−^ current [*I*_Cl(Ca)_]. The Courtemanche model was, together with Nygren et al., the earliest to be published.

The intracellular Ca^2+^ handling subsystem in the Courtemanche model is based on the guinea pig ventricular model of (22), whereas the Grandi model uses the formulation of the rabbit ventricular Ca^2+^ handling previously described (44). In the Maleckar model, the Ca^2+^ subsystem is described as in the rabbit atrial model previously published (21). Intermodel differences related to the number of cellular compartments are also seen. The Courtemanche model is simplest in this regard, consisting of the extracellular and intracellular spaces between which the transmembrane currents flow. The Maleckar model additionally comprises the cleft space outside of the sarcolemma, where local accumulation and depletion of ions is permitted. This cleft space acts as a buffer between the intracellular and extracellular ionic solutions and can be thought of as an effective extracellular space. The Grandi model consists of the intracellular, subsarcolemmal, and extracellular spaces, with the subsarcolemmal space bridging the intracellular and extracellular compartments and acting as an effective intracellular space in terms of ionic concentrations seen by transmembrane currents.

As a result of these differences, the Courtemanche model generates a spike-and-dome AP morphology with a slowly decaying intracellular CaT, whereas the Maleckar model produces a triangular AP with a larger CaT amplitude and faster decay. The Grandi model produces an AP that is triangular but lower in amplitude than in the other models, and its CaT is slower to rise and decay.

#### Modifications to the generic models to capture two peaks in I_CaL_ density.

Figure 1, *D* and *E*, shows a comparison of *I*_to_, *I*_Kur_, *I*_K1_, and *I*_CaL_ densities recorded ex vivo with those yielded by the three generic in silico models. Simulated current-voltage curves of K^+^ repolarization currents in all three models qualitatively follow experimental recordings. In silico, *I*_CaL_ density is maximized at 10 mV in the Courtemanche and Maleckar models and at 0 mV in the generic Grandi model (7, 15, 23). Analysis of ex vivo *I*_CaL_ voltage-clamp data revealed two groups of cells with *I*_CaL_ density maximized at 10 and 20 mV, respectively (Fig. 1*E*). To capture experimental variability in *I*_CaL_ peak density location, L-type Ca^2+^ channel gating was modified in all three generic models. Parameters that control the membrane potential maximizing *I*_CaL_ density include half-potentials of activation (*V*_act_) and inactivation (V_inact_). In the generic Courtemanche, Maleckar, and Grandi models, *V*_act_ takes values of −10.0, −9.0, and −9.0 mV and V_inact_ is −28.0, −27.4, and −30.0 mV, respectively. For each of the three models, a parameter sweep was conducted where both *V*_act_ and *V*_inact_ were scaled by factors ranging from −2.0 to +2.0 of their generic model values in steps of 0.2. A change of *V*_act_ to 0.0 mV in the Grandi model shifted the peak *I*_CaL_ density to 10 mV, whereas altering *V*_act_ to 4.0, 3.6, and 10.8 mV in Courtemanche, Maleckar, and Grandi models yielded peak *I*_CaL_ density at the transmembrane potential of 20 mV (Fig. 1*E*).

### SMC Sampling for Experimentally Calibrated Populations of Models

The SMC approach imposes the experimentally motivated calibration constraints in distinct stages (this contrasts with LHS, where all experimentally motivated constraints can be applied simultaneously to all models within the candidate population). At each stage, SMC requires that the values of a specific AP biomarker produced by all models in the population fall within the experimentally permitted ranges for all pacing frequencies. Introduction of a new set of constraints can potentially result in sharp reductions in the regions of the parameter space that are viable. To “soften” this effect, constraints are not introduced in a strict fashion.

Expressed in simplified form, the algorithm proceeds as follows: *1*) initializes particles randomly throughout the search region of the parameter space (base values of model parameters ± 100%); *2*) tests each particle for how close it falls to the acceptable range of values for the first biomarker at all pacing frequencies (this distance is the 2-norm of the vector of distances away from the acceptable range for each biomarker at each pacing frequency); *3*) takes all particles that fully satisfy the constraint for all pacing frequencies (distance of 0) and then additional particles selected per increasing distance until at least one-half of the particles have been chosen (the distance from the acceptable range of values of the furthest particle becomes the new acceptable limit); *4*) copies the remaining particles (those outside the new acceptable limit) on random members of the set of particles that were chosen; *5*) performs several iterations (here 5) of Markov chain Monte Carlo on the copied particles to move them to unique locations in the parameter space that are still within the currently acceptable distance away from the constraint; *6*) repeats *steps 2* to *5* until all particles correspond to models that produce values for the current biomarker that fall within the desired ranges for each pacing frequency; and *7*) repeats *steps 2* to *6* for each biomarker, ensuring that, during trialed updates, no particles move to locations that would break any of the previously satisfied constraints.

The method is explained in full mathematical detail (9). Following the approach described therein, the Markov chain Monte Carlo steps are performed using a jumping distribution constructed by fitting a three-component Gaussian mixture model to the set of particles selected before each copy step.

### Computational Stimulation Protocols

#### Computational AP protocol.

We assumed that ionic concentrations in the pipette and extracellular solutions used in experiments corresponded to the intracellular and extracellular concentrations in the models. The intracellular Na^+^ and K^+^ and extracellular Na^+^, K^+^, and Ca^2+^ concentrations were held constant in time. Each model was stimulated for 100 beats at every frequency. Stimulus current duration and amplitude were adjusted in the models to capture the typical amount of charge injected in the cells ex vivo (60 pC). In the simulations with the candidate population based on the Maleckar model, the intracellular K^+^, Na^+^, and Mg^2+^ and effective extracellular K^+^, Na^+^, and Ca^2+^ concentrations were set to 130, 10, 2, 5.4, 140, and 1.8 mM, respectively. In the simulations with the populations based on the Grandi model, the effective intracellular K^+^, Na^+^, Cl^−^, and Mg^2+^ and extracellular K^+^, Na^+^, Ca^2+^, and Cl^−^ concentrations were set to 130, 10, 14, 2, 5.4, 140, 1.8, and 151.4 mM, respectively. For the candidate populations derived from the Courtemanche model, the intracellular K^+^ and Na^+^ and extracellular K^+^, Na^+^, and Ca^2+^ concentrations were set to 130, 10, 5.4, 140, and 1.8 mM, respectively. In an attempt to better mimic experimental conditions ex vivo, the (effective) intracellular Na^+^ and K^+^ and (effective) extracellular Na^+^, K^+^, and Ca^2+^ concentrations were held constant in time. In the candidate populations based on the Courtemanche model, this meant clamping the intracellular K^+^ and Na^+^ (the model lacks the effective extracellular space of the Maleckar model or the effective intracellular space of the Grandi model). In the populations based on the Maleckar model, the effective extracellular concentrations of K^+^, Na^+^, and Ca^2+^ and the intracellular concentrations of K^+^ and Na^+^ were clamped. In the candidate populations based on the Grandi model, the Na^+^ concentrations in the intracellular, effective intracellular, and cleft spaces (the latter being a part of the Ca^2+^-handling subsystem) were clamped.

#### Computational voltage-clamp protocols.

In the voltage-clamp simulation protocol designed to elucidate *I*_to_, the holding potential of −80 mV was maintained for 200 ms before the prestep to −40 mV for another 200 ms. After this, the transmembrane potential was set to the desired value (between −70 mV and 50 mV in 10-mV increments, with the exception of the increment between −10 and 10 mV, which was set to 0.0001 mV) for 700 ms followed by the voltage step to −40 mV for 200 ms and then to −80 mV for another 200 ms.

In the voltage-clamp simulation protocol designed to elucidate *I*_Kur_, the holding potential was set to −80 mV for 200 ms followed by a prestep to −35 mV for another 200 ms and then by the desired voltage step (between −35 and 45 mV in 10-mV increments) for 500 ms. After this, the membrane potential was set to −35 mV and then to −80 mV for 200 ms each. As in experiments, *I*_sus_ was measured as the amplitude of the current remaining at the end of the pulse relative to the zero current level at the end of the prestep to −35 mV. To mimic the block of *I*_Kur_ by 4-AP, values of *G*_Kur_ were set to zero in the relevant simulations, and *I*_Kur_ was determined as the difference in the total transmembrane current *I*_sus_ elucidated in the absence or presence of the simulated *I*_Kur_ block by 4-AP.

In the simulations of *I*_to_ and *I*_Kur_, the inhibitory effect of Cd^2+^ on *I*_CaL_ was modeled by setting *G*_CaL_ = 0 (32). The (effective) intracellular K^+^, Na^+^, Mg^2+^, and Cl^−^ concentrations were set to 140, 10, 2, and 24 mM, whereas the (effective) extracellular K^+^, Na^+^, Ca^2+^, and Cl^−^ concentrations were set to 5.4, 140, 1.8, and 152.4 mM, respectively, where applicable. The temperature was set to 310.15 K.

In the protocols for measuring *I*_CaL_, the holding potential was set at −40 mV for 125 ms followed by a voltage step to the desired voltage (between −50 and 50 mV in 10-mV increments, with the exception of the increment between −10 and 10 mV, which was set to 0.0001 mV) for 250 ms. After this, the transmembrane potential was held at −40 mV for another 125 ms. The temperature was set to 310.15 K.

In the simulations of *I*_CaL_, all transmembrane K^+^ currents (including the background K^+^ current in the Courtemanche model and the junctional component of *I*_Ks_ in the Grandi model) were switched off by setting their conductances to zero to mimic experimental inhibition of K^+^ channels by cesium ions (32); Na^+^ currents (including the background currents) were also switched off because of the absence of Na^+^ in the pipette and extracellular solutions in *I*_CaL_ voltage-clamp experiments. The absence of Na^+^ and K^+^ in experimental solutions was mimicked by setting the (effective) intracellular and extracellular Na^+^ and K^+^ concentrations to 0.0001 mM. The (effective) extracellular Ca^2+^ and Cl^−^ concentrations were set to 1.8 and 151.4 mM, whereas the (effective) intracellular Mg^2+^ and Cl^−^ concentrations were set to 7 and 16 mM. Exceptions here were the Grandi-based models, where intracellular Mg^2+^ was set to 5.5 mM, since higher values of this concentration resulted in lack of numerical stability.

In the voltage-clamp simulation protocol designed to elucidate *I*_K1_, the holding potential was set to −80 mV for 50 ms followed by a prestep to −100 mV for another 50 ms and then by a voltage ramp from −100 to +40 mV over the next 1,200 ms in fixed steps of 0.1167 mV/ms. After this, the membrane potential was set to −50 mV for 100 ms and then to −80 mV for 550 ms. The inhibitory effect of Ba^2+^ on *I*_K1_ was mimicked by setting *G*_K1_ = 0 in the relevant simulations, and *I*_K1_ was determined by subtraction of the total transmembrane current traces in the presence or absence of this simulated block. Temperature was set to 295.15 K. The (effective) extracellular concentrations of K^+^, Na^+^, Ca^2+^, and Cl^−^ were set to 20, 120, 1.8, and 145.6 mM where applicable. The (effective) intracellular K^+^, Na^+^, Mg^2+^, and Cl^−^ concentrations were set to 120, 8, 5, and 52 mM, respectively.

In all voltage-clamp simulations, the (effective) intracellular K^+^ and Na^+^ and (effective) extracellular K^+^, Na^+^, and Ca^2+^ concentrations were held constant in time. The transmembrane potential was also held constant in time (i.e., d*V*/d*t* = 0) with the exception of changes corresponding to the desired voltage steps. The stimulus current amplitude was set to zero in all simulations.

### Numerical Methods and Data Analysis

The numerical simulations were performed using Chaste (31), an open-source software framework for modeling in computational biology. The CellML implementation of the single-cell human atrial electrophysiological model by Courtemanche et al. (7), Maleckar et al. (23), and Grandi et al. (15) was used. The ordinary differential equations were integrated with the software package CVODE (https://computation.llnl.gov/casc/sundials/). A backward differentiation formula method used to integrate the differential equations had the absolute and relative error tolerances set to 10^−9^ and 10^−10^. Electrophysiological properties were output every 1 ms. Postprocessing of AP traces to yield AP properties was performed in Chaste. Data analysis was performed with Matlab. Graphs were plotted using Matlab.
